# Nonrigid Registration Regularized by Shape Information: Application to Atlas Construction of Cardiac CT Images

**DOI:** 10.1371/journal.pone.0130730

**Published:** 2015-06-25

**Authors:** Yunfei Zha, Xuesong Lu, Li Wang, Rongqian Yang, Shanxing Ou, Dong Xing, Defeng Wang

**Affiliations:** 1 Department of Radiology, Renmin Hospital of Wuhan University, Wuhan 430060, P. R. China; 2 College of Biomedical Engineering, South-Central University for Nationalities, Wuhan 430074, P. R. China; 3 Department of Infection Control, Renmin Hospital of Wuhan University, Wuhan 430060, P. R. China; 4 School of Materials Science and Engineering, South China University of Technology, Guangzhou 510006, P. R. China; 5 Radiology Department, Guangzhou General Hospital of Guangzhou Military Area Command, Guangzhou 510010, P. R. China; 6 Research Center for Medical Image Computing, Department of Imaging and Interventional Radiology, The Chinese University of Hong Kong, Shatin, New Territories, Hong Kong, China; 7 Shenzhen Research Institute, The Chinese University of Hong Kong, Shenzhen, China; The First affiliated Hospital of Xi’an Jiaotong University, CHINA

## Abstract

Cardiac atlases play an important role in the computer-aided diagnosis of cardiovascular diseases, in particular they need to deal with large and highly variable image datasets. In this paper, we propose a new nonrigid registration algorithm incorporating shape information, to produce comprehensive atlases. For one thing, the multiscale gradient orientation features of images are combined to form the construction of multifeature mutual information. Additionally, the shape information of multiple-objects in images is incorporated into the cost function for registration. We demonstrate the merits of the new registration algorithm on the 3D data sets of 15 patients. The experimental results show that the new registration algorithm can outperform the conventional intensity-based registration method. The obtained atlas can represent the cardiac structures more accurately.

## Introduction

Cardiovascular diseases are now the most significant cause of death in China [1]. Their early diagnosis and treatment is crucial in order to reduce mortality and to improve patients’ quality of life. In recent years, some non-invasive imaging modalities are establishing the importance of high resolution imaging of the cardiovascular system. Among them, Computed Tomography(CT) has been widely used in not only the acquisition of 3D images which describe the cardiac anatomy but also the acquisition of 3D+time image sequences which describe the cardiac anatomy as well as function [2]. Both the multislice CT(MSCT) and dual-source CT(DSCT) have a demonstrated high sensitivity and specificity in diagnoses [3, 4].

Cardiac atlases play an important role in computer-aided diagnoses of angiocardiopathy [5]. Their abilities mainly focus on three profiles: First of all, the patient-specific biomarkers can be automatically extracted. Secondly, the multi-modal data can be integrated into a unified space for visualization purposes. Thirdly, the patient-specific models can be generated for individualized simulations of alternative treatment scenarios.

Recently, some approaches using the image registration technique have been developed for the volumetric-based modeling of the heart [6]. Frangi et al. proposed a novel method for the generation of 3D shape landmarks, in order to construct statistical shape models of the heart [7]. It can treat multiple-part structures and requires less restrictive assumptions on the structure’s topology. Perperidis used voxel-based atlases of images to generate surface-based training data for a 3D+time statistical model [8]. Another 3D+time statistical model of the cardiac shape was based on a direct bilinear decomposition of the surfaces in a training set, using higher order singular value decomposition [9]. A construction method for a 4D atlas of the human heart using cardiac MR imaging was proposed [10]. The probabilistic atlas captures the cardiac anatomy and function of a healthy heart. A construction framework of a detailed atlas based on a spatial-temporal statistical model of the human heart was presented [11]. It uses spatial normalization based on a non-rigid image registration to synthesize a population mean image and establish the spatial relationships between the mean and the subjects in the population.

However, it is possible for largely variable image datasets to achieve a superior atlas without the statistical shape models of organs. In this paper, we propose a novel nonrigid registration algorithm for cardiac atlas construction. Multifeature mutual information incorporating multiscale gradient orientation features is employed as a similarity metric. The shape information of the object instead of the statistical shape model is incorporated into the cost function of registration as a regularization term. The experiments performed on the 3D+time cardiac data set of DSCT show that the new algorithm can achieve high registration accuracy. The synthetic mean atlas can represent the anatomical variety of cardiac structures more accurately.

## Materials and Methods

### Ethics Statement

This study was reviewed and approved by the ethics committee of Guangzhou General Hospital of Guangzhou Military Area Command. Informed written consents have been obtained from the participants.

### 2.1 Transformation Model

The goal of image registration in cardiac CT is to find the optimal transformation *T*:(*x*,*y*,*z*)↦(*x*′,*y*′,*z*′), which maps any point in the moving images *I*:(*x*,*y*,*z*) into its corresponding point in the reference image *I*(*x*′,*y*′,*z*′). In this work, we adopt a combined transformation T:

$$T\left(x,y,z\right)=T_{global}\left(x,y,z\right)+T_{local}\left(x,y,z\right)$$ (1)

The global transformation *T*_*global*_ is affine model, and the local transformation *T*_*local*_ is a free-form deformation(FFD) model based on B-splines [12]. The affine registration result is considered as the initial parameters for registration using the FFD model.

### 2.2 Multifeature Mutual Information

Normalized mutual information(MI) is generally calculated on the image intensities only, whereas multifeature mutual information(α-MI) can measure higher-dimensional signals through graph theory [13]. The fixed image is denoted as *F* and the moving image *M*. The aim of registration is to search the optimal parameter *μ* of transformation *T*_*μ*_(*x*) between *F* and *M*. Let *z* ^*f*^(*x*_*i*_) be the feature vector of *F* at a point *x*_*i*_, and *z* ^*m*^(*T*_*μ*_(*x*_*i*_)) that of *M* at the transformed point *T*_*μ*_(*x*_*i*_). Let *z* ^*fm*^(*x*_*i*_,*T*_*μ*_(*x*_*i*_)) be the concatenation of the two feature vectors: [*z* ^*f*^(*x*_*i*_),*z* ^*m*^(*T*_*μ*_(*x*_*i*_))]. Three k-nearest neighbor(KNN) graphs $\Gamma_{i}^{f}$, $\Gamma_{i}^{m}\left(\mu \right)$, and $\Gamma_{i}^{fm}\left(\mu \right)$ are respectively constructed by the total distance of a feature vector *z* to its *k* nearest neighbors:

$$\Gamma_{i}^{f}=\sum_{p=1}^{k}\left\|z^{f}\left(x_{i}\right)-z^{f}\left(x_{ip}\right)\right\|$$ (2)

$$\Gamma_{i}^{m}\left(\mu \right)=\sum_{p=1}^{k}\left\|z^{m}\left(T_{\mu }\left(x_{i}\right)\right)-z^{m}\left(T_{\mu }\left(x_{ip}\right)\right)\right\|$$ (3)

$$\Gamma_{i}^{fm}\left(\mu \right)=\sum_{p=1}^{k}\left\|z^{fm}\left(x_{i},T_{\mu }\left(x_{i}\right)\right)-z^{fm}\left(x_{ip},T_{\mu }\left(x_{ip}\right)\right)\right\|$$ (4)

So α-MI is defined as:

$$\alpha -MI\left(\mu \right)=\frac{1}{\alpha -1}\text{log}\frac{1}{N^{\alpha }}\sum_{i=1}^{N}{\left(\frac{\Gamma_{i}^{fm}\left(\mu \right)}{\sqrt{\Gamma_{i}^{f}\Gamma_{i}^{m}\left(\mu \right)}}\right)}^{2\gamma }$$ (5)

with *γ* = *d*(1-*α*), and 0<*α*<1. *z* ^*f*^(*x*_*ip*_), *z* ^*m*^(*T*_*μ*_(*x*_*ip*_)), and *z* ^*fm*^(*x*_*ip*_,*T*_*μ*_(*x*_*ip*_)) are the *p*th nearest neighbor of *z* ^*f*^(*x*_*i*_), *z* ^*m*^(*T*_*μ*_(*x*_*i*_)), and *z* ^*fm*^(*x*_*i*_,*T*_*μ*_(*x*_*i*_)), respectively. *N* is the number of sample points.

It is crucial to select representative features for registration using an α-MI. A Cartesian image set with 15 features was chosen for nonrigid registration by cervical MRI [14]. These features consist of *L*, *g* ^*T*^ *g*, *g* ^*T*^ *Hg*, *g* ^*T*^ *HHg*, *tr*(*H*), *tr*(*HH*), and *tr*(*HHH*). Here, *L* is the image intensity, g = *∂L/∂xg* the spatial derivative, *H* is the Hessian of *L*, and *tr*(·) denotes the matrix trace. They are all computed using Gaussian derivatives at scale *σ*. In fact, the boundaries of some substructures in medical images often carry significant information. Although the gradient of the boundaries might not have the same magnitude in some cases, the gradient orientation should be the same [15, 16]. In this paper, we combine multiscale gradient orientation features with Cartesian features to compute the α-MI. Assuming that the gradient vector for each voxel is defined as (*v*_1_,*v*_2_,*v*_3_), the orientation described by two angles *θ* and *φ* is given by:

r=v12+v22+v32, θ=arccos(v3r),φ=arctan(v1v2) (6)

Finally, these features consist of the original intensity image, Cartesian features at scales *σ* = 1,2, and a corresponding gradient orientation at scales *σ* = 1,2. They are normalized to have a zero mean and unit variance.

### 2.3 Cost Function

In atlas construction, some methods involve the statistical shape model. In some cases, an accurate registration could perform better than the existing statistical shape models. In this section we incorporate shape information into nonrigid registration. The cost function comprises two competing goals, followed by:

$$C=-C_{\alpha -MI}+\omega C_{P}$$ (7)

The first term is the α-MI, while the second term *C*_*P*_ represents the cost related to the consistency of shape points. A weighting factor *ω*(0.01≤*ω*≤0.1) is used to balance the two terms.

The extraction of corresponding shape points is very similar to [7]. Firstly, the marching cubes [17] algorithm is applied to generate a dense triangulation of the boundary isosurfaces in binary fixed image. Then the decimation process can be implemented in order to reduce the amount of triangular nodes. We use the method by Schroeder et al. [18], and specify a target decimation rate (96%) with respect to the original mesh. These nodes in the decimated triangulation will form the landmarks of the shape. Finally, the shape points of the fixed image can be propagated to the moving image through a volumetric registration of the two binary images using a kappa statistics metric [19].

A parametric representation of N shape point pairs is denoted as {(*p*_*fi*_,*p*_*mi*_):*i* = 1,2,…,*N*}, where *p*_*fi*_ = [*x*_*fi*_,*y*_*fi*_,*z*_*fi*_] in the fixed image and *p*_*mi*_ = [*x*_*mi*_,*y*_*mi*_,*z*_*mi*_] in the moving image. So the penalty term *C*_*P*_ is defined as:

$$C_{p}=\frac{1}{N}\sum_{i=1}^{N}\left\|p_{mi}-T_{\mu }\left(p_{fi}\right)\right\|$$ (8)

where *T*_*μ*_(·) is the transformation and ||·|| is the Euclidean distance. For speed and efficiency, most of free-form registration methods based on B-splines use gradient-based optimizers. Consequently, the derivatives of the penalty term with respect to the transform parameters are required. The derivative of *C*_*P*_ reads:

$$\frac{\partial C_{p}}{\partial \mu }=-\frac{1}{N}\sum_{i=1}^{N}\frac{1}{\left\|p_{mi}-T_{\mu }\left(p_{fi}\right)\right\|}\left(p_{mi}-T_{\mu }\left(p_{fi}\right)\right)\frac{\partial T}{\partial \mu }\left(p_{fi}\right)$$ (9)

### 2.4 Atlas Construction

Atlas construction is a different and complicated topic outside the scope of image registration. In this work, we construct a simple atlas to test the performance of the proposed registration approach. Similar to Ref. [20], this simple atlas can be built without a statistical shape model, because it is assumed that a nonrigid registration incorporating shape information could perform better when applied to highly variable cardiac datasets. This atlas can be produced from a selected reference space such as the mean of a group of cardiac CT images.

**Fig 1:**
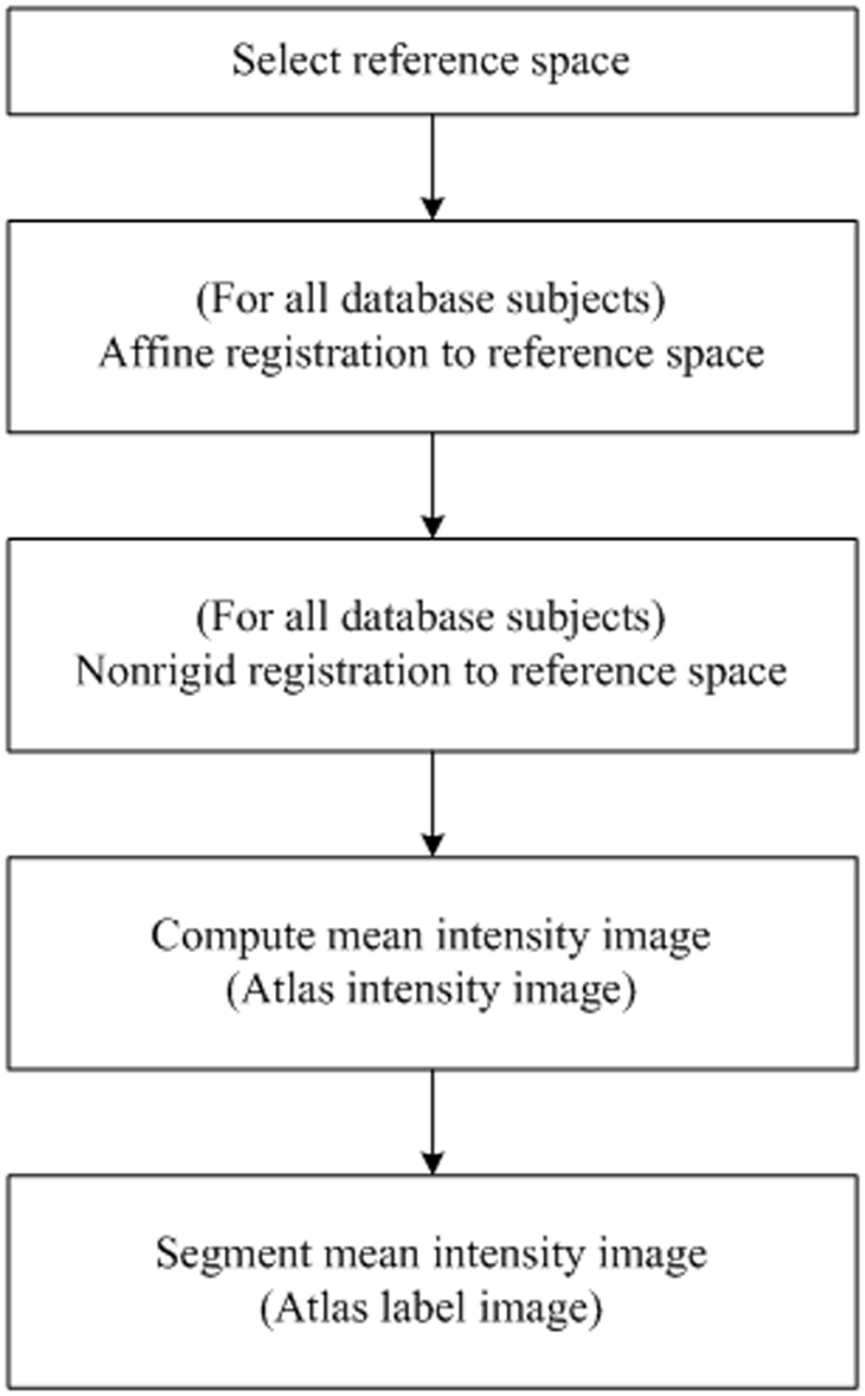
Flow diagram for atlas construction.

In practice, a reference space is initially selected from a population of images. The other images are then registered to this reference space. A mean intensity image, referred to as the atlas intensity image, can be computed from this set of registered images. The labeling of each anatomical region of the reference space has the corresponding segmentation information of the atlas, referred to as the atlas label image. The process is outlined in Fig 1.

## Experiments

The nonrigid registration as described above was implemented in Elastix version 4.6 [21]. The extraction algorithm of shape points is based on the Visualization Toolkit(VTK) [22]. All programs were run on a Windows computer with an Intel Dual Core 3.40 GHz CPU and 16.0 GB memory. The running time for each registration is about 2 hours.

### 3.1 Data

**Fig 2:**
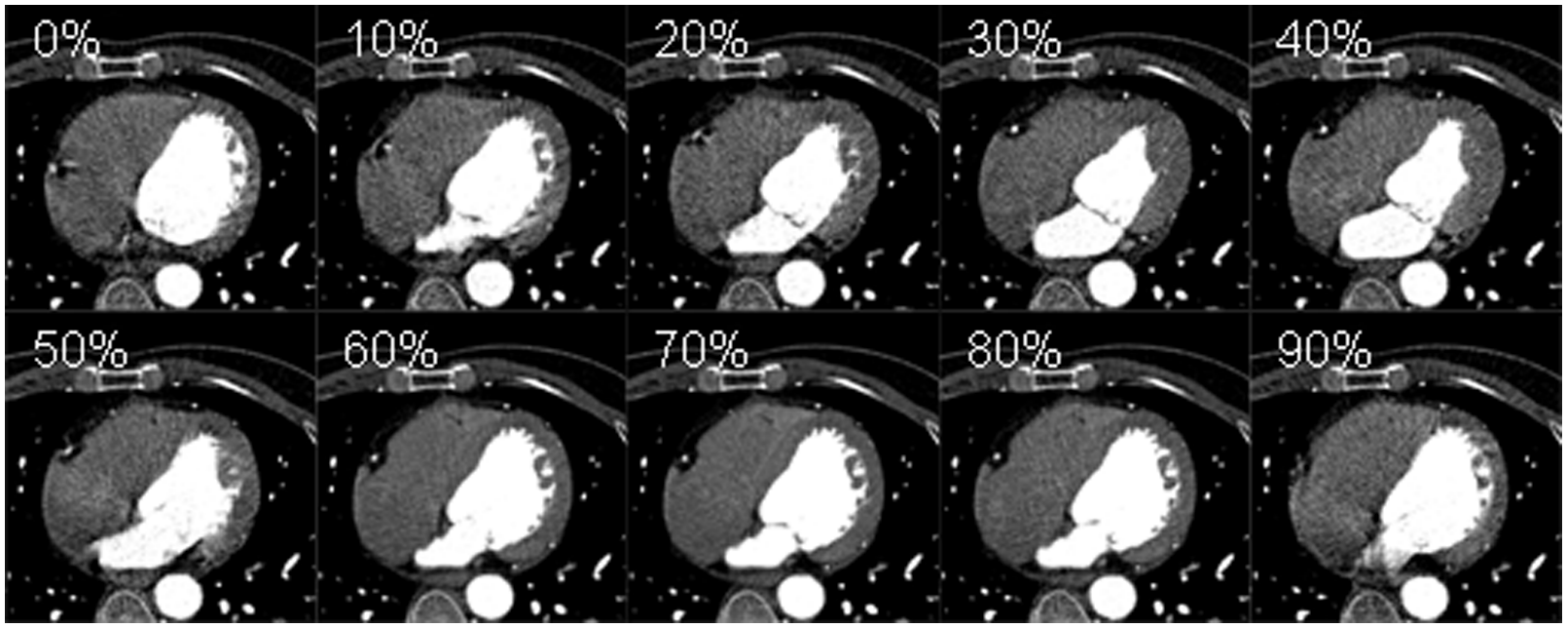
The sequence images of a patient at ten time phases.

The cardiac data was acquired with a dual source CT scanner (Siemens Somatom Definition, Germany). Fifteen patients were scanned. Each patient was scanned at twenty-one time phases. Fig 2 shows the sequence image of a patient at ten time phases. As the goal of this work is to test the performance of the proposed approach, the first time phase occurring during the diastole of all patients was used in the experiment. The image dimensions were 512×512×254 voxels of size 0.348×0.348×0.5*mm*. All the data are publicly available through http://figshare.com/articles/cardiac_ct_data_15sets/1379059.

**Fig 3:**
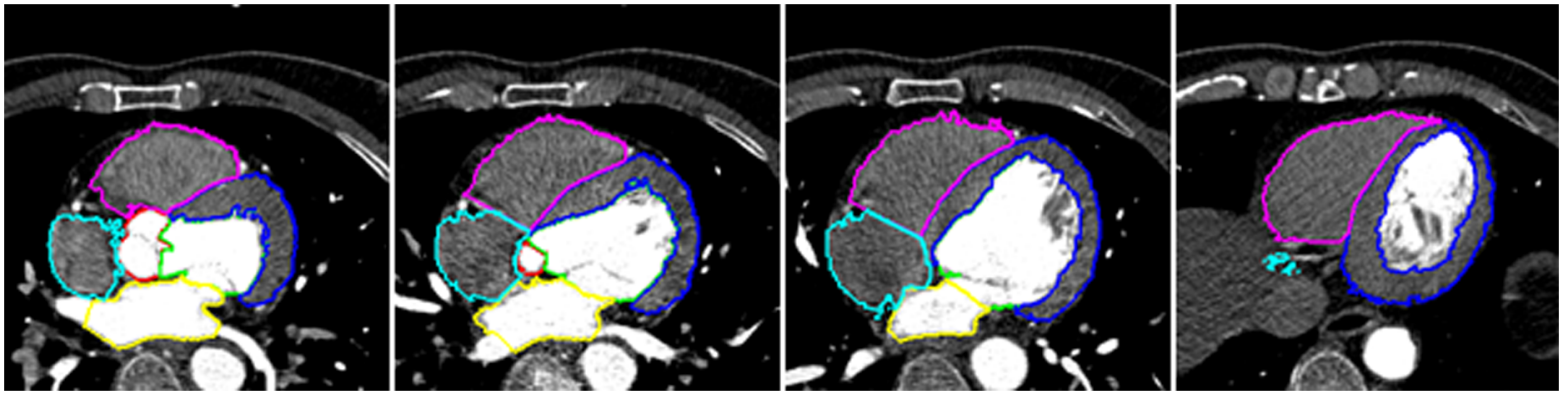
Manual segmentation results of registration images.

Manual segmentations of AO(Aorta), LV(Left Ventricle), LVM(Left Ventricle Myocardium), LA(Left Atrium), RV(Right Ventricle), and RA(Right Atrium) were available for each image. They were either used to extract shape points or considered as the gold standard. The manual segmentation was completed by either a clinician or a research associate with an expert knowledge of heart anatomy. Some of them are displayed in Fig 3. The baseline of the experiment is a traditional mutual information method.

### 3.2 Evaluation Method

To evaluate the registration quality, automatic segmentation results of the fixed images were created by transforming the manual segmentation of the moving images, with transformation of the registration results. The Dice Similarity Coefficient (DSC) [23] as a measure of overlap, was calculated between the automatic segmentations and manual segmentations of the fixed image. *DSC* = 0 indicates no overlap while *DSC* = 1 indicates perfect agreement. The DSCs are presented in box-and-whisker plots.

To compare registration results of the two algorithms, two-sided Wilcoxon tests [24] were carried out on the corresponding DSC values. A value of *p*<0.05 was regarded as a statistically significant difference.

### 3.3 Choice of Parameters

In order to validate the new algorithm, we randomly selected the image of the fourth patient as the fixed image, and images of the other patients as the moving images. An affine initial registration using the MI of intensities, only was performed before the nonrigid registration, to get a rough alignment. For the nonrigid registration of traditional MI and α-MI, a multiresolution scheme with five levels was employed. Gaussian smoothing was applied with scales *σ* = 16,8,4,2,1, but no downsampling to more accurately interpolate the moving image. As for the B-spline control points, the grid spacing of 80, 40, 20, 10, and 5 mm was applied to the five resolution levels, respectively. For the optimization procedure, *A* = 50, *τ* = 0.6, and *a* = 2000 were set. 1000 iterations were used. The number of samples was set to *N* = 5000.

For the α-MI, the *KD* trees; a standard splitting rule; a bucket size of 50; and an error bounding value of 10 was selected. The(*k* = 5) nearest neighbors were set while *α* = 0.99 was set. About 1500 shape point pairs were extracted from five surfaces(including AO, LVM, LA, RV, and RA) in manual segmentations. The balance coefficient *ω* = 0.05 was found to yield the best registration result.

## Results

**Fig 4:**
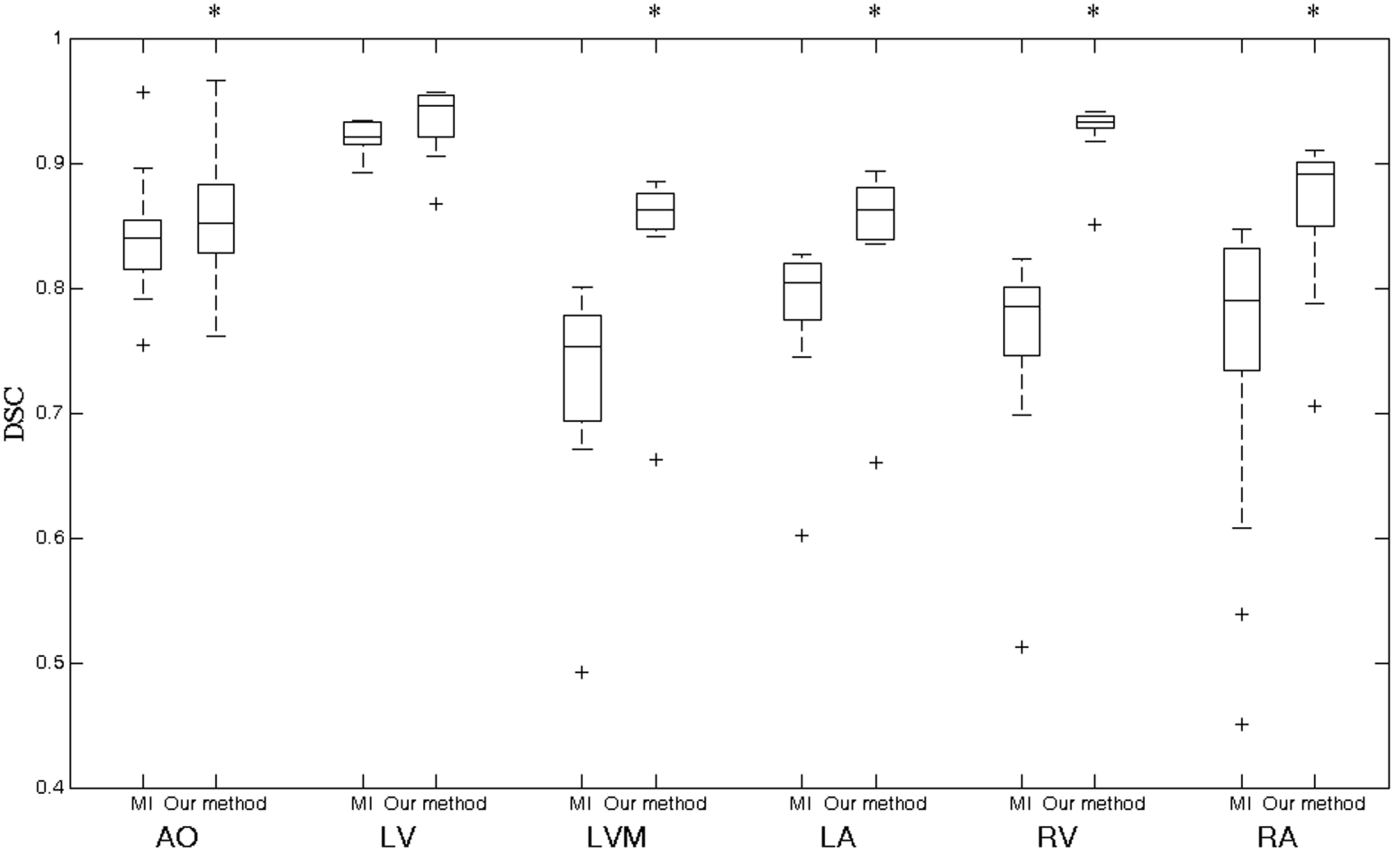
The comparison of registration accuracy using traditional MI and our method. A star indicates a statistical significant difference of the median DSC of the two methods.

In this section, the registration accuracy of traditional MI and our method was compared. Fig 4 shows the DSC results of registration for AO, LV, LVM, LA, RV, and RA. The improvement of the average DSC for AO, LVM, LA, RV, and RA are found to be significant. For LV, the improvement of accuracy is minor. The overall mean and standard deviation of the DSCs in the experiments are summarized in Table 1.

**Table 1:** A summary of the DSC value for six cardiac components.

Structure	Method	DSC
AO	MI	0.8417 ± 0.0025
	Our method	0.8524 ± 0.0027
LV	MI	0.9199 ± 0.0002
	Our method	0.9357 ± 0.0006
LVM	MI	0.7281 ± 0.0061
	Our method	0.8502 ± 0.0031
LA	MI	0.7853 ± 0.0035
	Our method	0.8169 ± 0.0200
RV	MI	0.7572 ± 0.0063
	Our method	0.9280 ± 0.0005
RA	MI	0.7477 ± 0.0158
	Our method	0.8655 ± 0.0033

**Fig 5:**
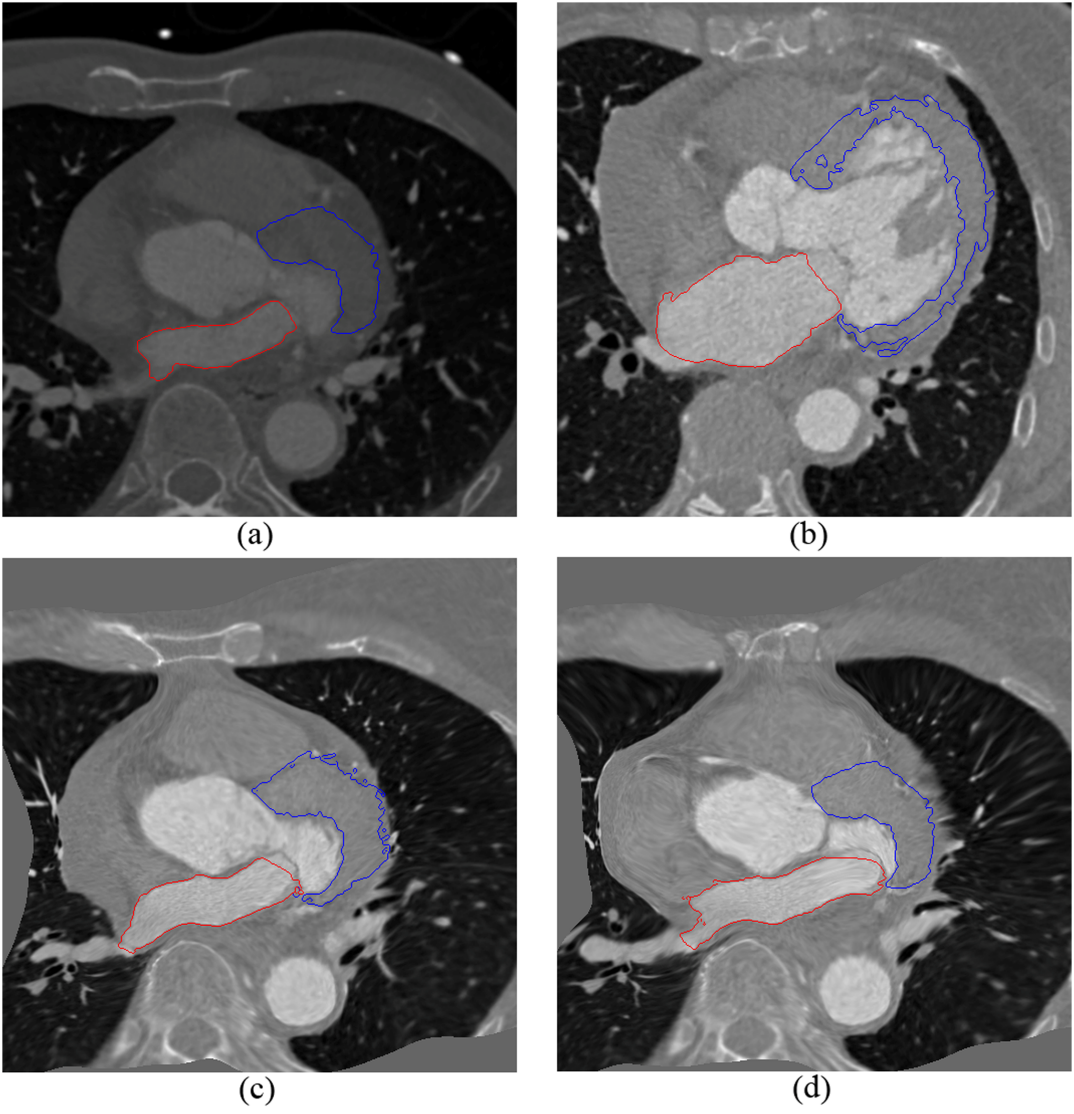
Example of the registration result: the red contour surround LA region, the blue contour surround LVM region. (a) The fixed image. (b) The moving image. (c) The deformed moving image using MI. (d) The deformed moving image using our method.

**Fig 6:**
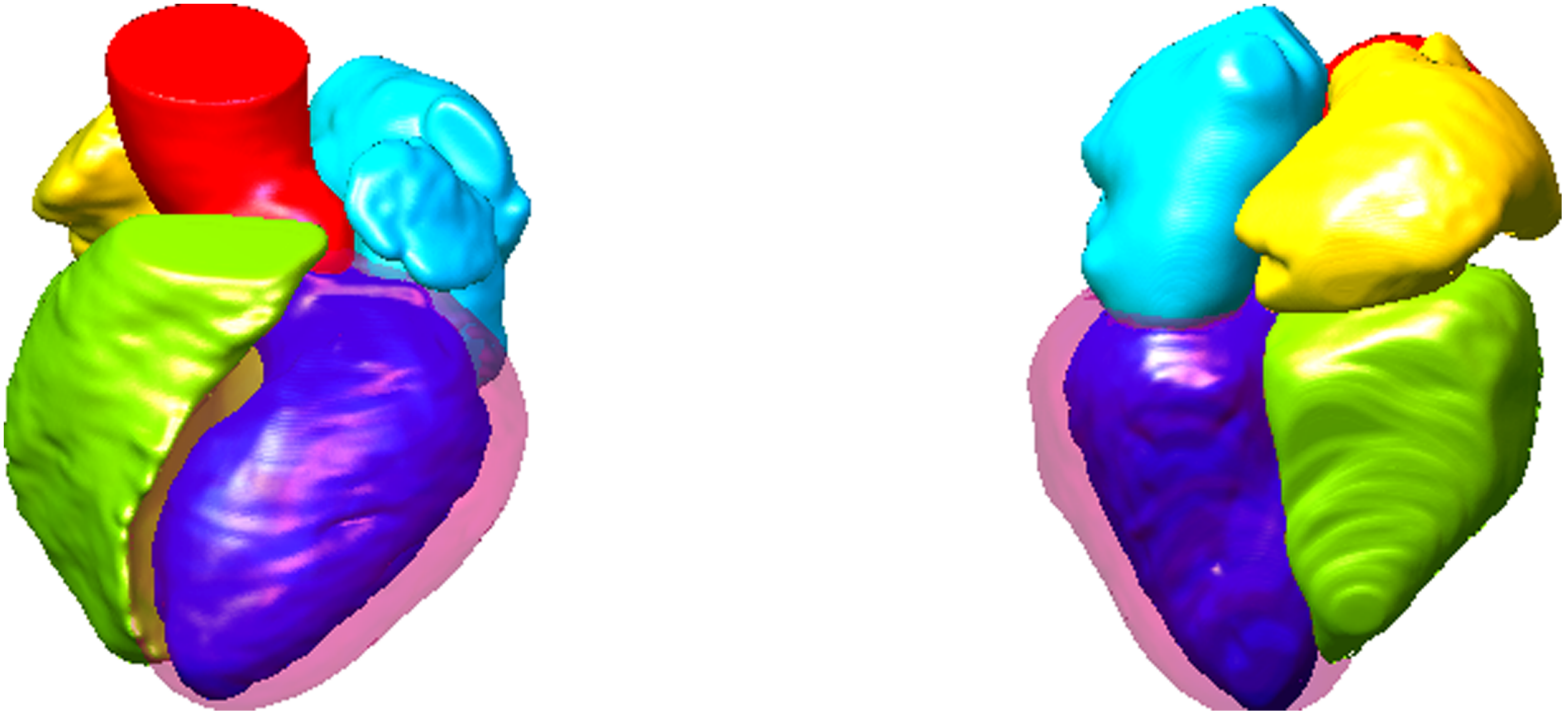
Two views of atlas mesh corresponding to the synthesized mean image. Different colors indicate different structures.

In Fig 5, a typical example of the registration result using MI (c) and our method (d) is illustrated. The LA and LVM of the fixed (a) and moving (b) image are very different. It can be observed that our method can achieve better alignment with the reference image than using the MI method. Fig 6 shows two views of the atlas mesh, corresponding to the synthesized mean image by registration. They can clearly display the six structures of cardiac images.

## Discussion

In this paper, we set out to improve the accuracy of a nonrigid registration for cardiac atlas construction. Multiscale gradient orientation features of images are extracted to construct multifeature mutual information. Additionally, the shape information of multiple-objects in images is incorporated into the cost function of registration. We have demonstrated the atlas construction using this method over a population of 15 subjects. We validated the atlas through a measure of registration accuracy. It was shown that the proposed method outperforms traditional MI.

There are some limitations in this work. Firstly, the computational burden of this method needs to be decreased for use in the clinic, although the dimensionality of features is not high. In this case it might be possible to parallelize the important parts of the algorithm making use of multiple processors. Secondly, the number of structures in the atlas needs to be expanded. The registration accuracy probably depends on the shape points of cardiac structures. The improvement of AO and LV structures is less than that for other structures. Ascertaining the reason for this provides an additional direction for future research. Finally, the subjects used in our experiments may not be enough to represent the cardiac atlas with sufficient diversity. More cases would make it more probable to display a wide diversity of morphologies and pathologies.

In future work, compensative techniques will be considered to deal with these limitations. The parallel computation such as using graphic processing units will be used to accelerate the registration process. We will also investigate the stability of the algorithm with cardiac structures in clinical practice.
